# The CX-DZ-II intelligent electronic stimulator for neck pain caused by cervical spondylosis: A two-center, randomized, controlled, and non-inferiority trial

**DOI:** 10.3389/fnins.2022.910574

**Published:** 2022-07-28

**Authors:** Liping Chen, Dehua Li, Jing Xu, Hao Liang, Ya Zhang, Yulan Ren, Fanrong Liang

**Affiliations:** ^1^College of Acupuncture Moxibustion and Tuina, Chengdu University of Traditional Chinese Medicine, Chengdu, China; ^2^Hospital of Chengdu University of Traditional Chinese Medicine, Chengdu, China; ^3^College of Chinese Classics, Chengdu University of Traditional Chinese Medicine, Chengdu, China

**Keywords:** electroacupuncture, neck pain, cervical spondylosis, randomized controlled trial, non-inferiority trial

## Abstract

**Background:**

Electroacupuncture (EA) has been commonly used for the management of neck pain caused by cervical spondylosis (NPCS); however, current electrical instruments have limitations on intelligence, digitalization, and visualization. The intelligent electronic stimulator (CX-DZ-II) is a digital device with an evidence-based diagnosis and treatment system. This study aimed to investigate the efficacy and safety of the CX-DZ-II intelligent EA instrument for NPCS.

**Materials and Methods:**

A total of 164 patients with NPCS [mean age (SD), 49.48 (13.47) years] were randomly assigned to receive 8 sessions (over 2 weeks) EA of the intelligent electronic stimulator (CX-DZ-II) or the regular electronic stimulator (SDZ-II). The primary outcome was the change of the visual analog scale (VAS) from baseline to 2 weeks of treatment. Secondary outcomes included mean scores of the VAS after each treatment in 1 week, responder rate, drug-usage rate of non-steroidal antipyretic analgesics (NSAAs), the occurrence rate of adverse events (AEs), proportions of apparatus with defect during treatment, and excellent rate of apparatus.

**Results:**

The intelligent electronic stimulator (CX-DZ-II) was non-inferior to the regular electronic stimulator (SDZ-II) for changes from baseline in the VAS [3.36 vs. 3.23, with a difference of 0.17 (95% CI, −0.36 to 0.69), *P* < 0.025 for non-inferiority]. No between-group differences were found in outcomes of VAS in 1 week, overall responders, and drug-usage rate of NSAAs. The defect rate and excellent rate of the instrument were similar in the CX-DZ-II and SDZ-II groups. Adverse events occurred in 9 (10.84%) patients in the CX-DZ-II group and 4 (5.00%) patients in the SDZ-II group.

**Conclusion:**

The intelligent electronic stimulator (CX-DZ-II) was non-inferior to the regular electronic stimulator (SDZ-II) in relieving neck pain. The intelligent electronic stimulator (CX-DZ-II) is a promising non-inferior alternative instrument for NPCS.

**Clinical Trial Registration:**

[https://clinicaltrials.gov/], identifier [NCT030 05301].

## Introduction

Cervical spondylosis (CS) is an age-related degenerative condition presenting structural or functional damage to the cervical spinal cord, nerve roots, and adjacent blood vessels. Worldwide, the prevalence of CS increases by year and affects more younger people. A previous study showed that engaging in mental work, high housework intensity, and sleep duration of less than 7 h/day were the main contributors to the incidence of CS (Lv et al., 2018). Neck pain (NP) is the most common complaint of patients with CS (Vogt et al., 2006). According to the guidelines from America, Netherlands, and Denmark, CS is one of the main causes of NP (Blanpied et al., 2017; Kjaer et al., 2017; Bier et al., 2018). In the 2017 global burden of disease study, the incidence of NP per 100,000 population was 806.6 and the years lived with disability from NP per 100,000 population was 352.0 (Safiri et al., 2020). With the increasing costs and the long-lasting disability associated with CS, conducting research on the effectiveness of interventions designed to prevent and treat CS is crucial (Kuo and Tadi, 2022). Currently, a range of non-surgical treatments for NP caused by CS has been recommended, such as manual therapy, exercise, psychological therapies, and acupuncture (Chinese Medical Association, 2007; Corp et al., 2021).

Acupuncture is a physical intervention used for various pain management. Electroacupuncture (EA) is a prevalent therapy of acupuncture, with the integrated effect of a manual needle and electrical stimulation from instruments. Currently, EA has been widely used for various pain diseases, including chronic non-specific low back pain, post-operative pain, musculoskeletal pain, and pain relief during colonoscopy (Comachio et al., 2020; Huang et al., 2021b; Joan Gan et al., 2021; Mao et al., 2021). Especially, findings of the previous meta-analysis indicated that acupuncture might be effective for NP and EA that may relieve even more pain (Seo et al., 2017; Huang et al., 2021c). EA blocks pain by activating a variety of bioactive chemicals through peripheral, spinal, and supraspinal mechanisms (Zhang et al., 2014).

Electric stimulators are indispensable devices for EA therapy. Since the 1950s, EA instruments with multiple presentation formats had been innovated for clinical treatment, such as wearable devices, single-acupoint electronic apparatuses, and apparatus equipped with manual acupuncture techniques (Hong et al., 2006; Liu et al., 2010; Shen et al., 2016; Feng et al., 2019). Among them, the regular electronic stimulator (SDZ-II) has been widely used in various conditions management and scientific research (Luo, 2014; Su et al., 2016; Huang et al., 2021a), with qualified parameters (Wang, 2019). According to a previous report, the SDZ-II electronic stimulator was also effective for NP relief for patients with CS (Tang et al., 2014). Nonetheless, similar to other devices, the SDZ-II electronic stimulator has deficiencies in parameter adjustment, intelligence, digitalization, and visualization (Yang and Yong, 2009; Liu et al., 2016).

The intelligent electronic stimulator (CX-DZ-II) is a new intelligent device with an evidence-based diagnosis and treatment system (Liang et al., 2017). It is equipped with a display terminal and then accurate settings such as pulse waveform, frequency, intensity, and treatment time that can be provided to users visually. More importantly, the information can also be uploaded instantly, if WiFi is available (Jia et al., 2016). Generally, it owns the advantages of accurate parameter adjustment, visual data storage, and remote data management. Therefore, this study aimed to assess the effect and safety of the intelligent electronic stimulator (CX-DZ-II) in comparison with the regular electronic stimulator (SDZ-II) for NP relief in patients with CS and then provide an alternative for CS management.

## Materials and methods

### Study design

This randomized, non-inferiority trial (NCT03005301) was performed at the Hospital of Chengdu University of Traditional Chinese Medicine and the West China Hospital, Sichuan University. The study protocol has been approved by the Sichuan Regional Ethical Review Committee, affiliated with Chengdu University of Traditional Chinese Medicine (Approval No. 2016XL-007).

The total observation period in this study was 2 weeks for each participant. Eligible participants were randomly assigned to receive EA from the CX-DZ-II intelligent electronic stimulator or the SDZ-II regular electronic stimulator. All the outcome measurements were completed at baseline and 2 weeks after randomization. In addition, the visual analog scales (VASs) for each participant were also assessed after each treatment in the first week.

### Participants

Patients with CS of neck type or nerve root type were recruited through outpatient clinics from April 2017 to August 2017.

The diagnostic criteria of CS were established according to the Clinical Guidelines for Diagnosis and Treatment: Pain (Chinese Medical Association, 2007): (1) CS of neck type: pain in the neck, the shoulder, and the occipitalia; limited cranial movement with tense muscles and trigger points; X-rays showed that there were changes in cervical curvature, and dynamic radiographs showed instability and loosening of the intervertebral joints. (2) CS of nerve root type: neck, shoulder, and back pain, and even radiation of arm pain to forearm and fingers; sensation of electric touch, numbness, and obvious hypoesthesia in the nerve root innervation area; results of Eaton test were positive; X-rays showed that there were changes in uncinate joint hyperplasia, bone spur formation, narrowing of intervertebral space, and changes and even loss of physiological radians.

Participants who met all the following inclusion criteria were enrolled in this study: (1) male or female between 18 and 75 years of age; (2) a history of recurrent episodes of NP (one or more episodes of NP per month lasting for more than 3 months); (3) the VAS score >3; (4) subject or his supervisor can comprehend the aims and process of this trial; (5) not participating in other trials or receiving other relevant treatments during the trial periods; and (6) willing to sign informed consent. Participants with any of the following conditions were excluded: (1) subject has acute neck trauma; (2) previous medical history of neck trauma treated by surgery, neurological deficit, congenital and developmental spinal disorders, systemic bone diseases, or systemic joint diseases; (3) diagnosis of carotid artery dissection; (4) unable to clearly perceive the pain or express their feelings; (5) infection in the acupoint region; (6) history of acupuncture treatment for NP in the previous week; (7) use of non-steroidal antipyretic analgesics (NSAAs) in the previous 3 days, need to use central analgesics or narcotic analgesics during the period of clinical trial, use of any ointments/medicinal liquors with functions of promoting blood circulation and easing pain, and use of oral and intravenous medicines aiming at opening blood vessels and providing nerve nutrition; (8) combinations of severe diseases, such as myocardial infarction, severe hepatic renal dysfunction, acute infectious diseases, malignant tumors, or severe mental disorders in the previous 12 months; (9) intolerance of acupuncture and EA treatment or allergy to acupuncture; (10) pregnancy or lactation; (11) participation in other clinical trials in the previous 3 months, and (12) unsuitability to this trial as judged by the investigators.

### Randomization and masking

Eligible participants were randomized according to a computer-generated randomization list in sealed, opaque envelopes and were divided into two groups: the CX-DZ-II and SDZ-II groups. The randomization was stratified by enrollment site in a block size of 4 with a 1:1 ratio. The randomization list was conserved by the physicians not participating in the study. Participants, acupuncturists, and outcome assessors were not blinded because of the obvious difference in operating interface, shape, and appearance of the two study instruments. Only statisticians were blinded to treatment allocation.

### Interventions

Disposable acupuncture needles (0.35 mm × 25 mm, 0.35 mm × 40 mm, Huatuo, Suzhou Hualun Medical Appliance), the CX-DZ-II intelligent electronic stimulator (Chengdu Chengxin High-tech Company, Chengdu, China), and the SDZ-II regular electronic stimulator (Suzhou Medical Appliance Factory, registration No. 20133370611) were used.

All the treatments were performed by licensed acupuncturists. Participants in the treatment group received EA at basic acupoints: Dazhui (GV14), bilateral Fengchi (GB20), Jianjin (GB21), and Jiaji (EX-B2). Manual acupuncture was also performed at arbitrary acupoints, which were chosen based on syndrome differentiation: for participants with wind-cold dampness Bi syndrome, bilateral Fengmen (BL12), and Waiguan (TE5) were used; for participants with phlegm stasis in channels syndrome, bilateral Quchi (LI11), Pishu (BL20), Fenglong (ST40), and Geshu (BL17) were used; for participants diagnosed with deficiency of qi and blood syndrome, bilateral Ganshu (BL18), BL20, and Zusanli (ST36) were used; for participants with deficiency of Gan (liver) and Shen (kidney) syndrome, bilateral Yanglao (SI6), BL18, Shenshu (BL23), and Taixi (KI3) were used. The depth of the inserted needles differed but was approximately 15–40 mm. Following needle insertion, lifting, thrusting, twisting, and rotating, with a frequency of 60–90 times per min were performed on all the needles to achieve the *deqi* sensation. Two paired electrodes from the CX-DZ-II intelligent electronic stimulator were attached transversely to the needle handles at bilateral GB20 and EX-B2. The EA stimulation lasted for 30 min, with a dilatational wave of 2/100 Hz and a current intensity within the patient’s tolerance. Participants in the CX-DZ-II group received a total of 8 sessions of treatments: 5 sessions (every day) in the first week and 3 sessions (every other day) in the second week.

Participants in the control group received EA from the SDZ-II regular electronic stimulator. In the SDZ-II group, acupoints selection, depth of needles, needle manipulation for *deqi*, and electrode placements were consistent with the CX-DZ-II group.

### Outcomes measurement

The primary outcome was the change in the VAS from baseline to the completion of treatment.

Secondary outcomes included: (1) mean scores of the VAS after each treatment in the first week; (2) responder rate of participants with at least 70% increase from baseline in the treatment score, which was assessed based on the table for lower lumbar vertebral diseases of the Japanese Orthopedic Association; (3) drug-usage rate of NSAAs during treatment; (4) the occurrence rate of adverse events (AEs); (5) proportions of apparatus with defect during treatment; and (6) excellent rate of apparatus, defined as the proportions of instrument assessment with a score <16 points. The scores of the instrument were evaluated after each treatment according to the predesigned operating performance scale (1–5 indicating extremely difficult to easy) (see Supplementary Table 1).

### Statistical analysis

Based on a pilot study using the SDZ-II regular electronic stimulator for NP coursed by CS, the decline in the VAS score after a course of treatment was 5.18 ± 1.06 (Tang et al., 2014); considering clinical experience, we set -0.53 as the non-inferior margin. Thus, 160 participants were needed to provide 80% power to detect a difference between groups in the VAS score declination after treatment at a one-sided significance level of 0.025, assuming a 20% loss in the dropout rate (Chen et al., 2020).

Outcomes were analyzed according to the intention-to-treat principle, defined as all the randomized participants with baseline data receiving at least one treatment. The primary outcome was also assessed based on the per-protocol (PP) population, defined as all randomized participants without major protocol violations. The primary outcome was assessed using a one-tailed test at a significance level of 0.025, while the secondary outcomes used a two-tailed test at a significance level of 0.05. For the change in mean scores of the VAS after treatment, the *t*-test was used. The mean VAS score after each treatment was analyzed using repeated-measures ANOVA, setting group, time, and the interaction between group and time as fixed effects and center as covariates. For responder rate, the Cochran–Mantel–Haenszel test, stratified by site, was used to test a hierarchical comparison between groups. Drug-usage rate, the occurrence rate of AEs, defect rate, and excellent rate of the instrument between the two groups were compared using the chi-squared test or Fisher’s exact test.

Missing data for the primary outcome were imputed from the last observation carried forward. For secondary outcomes, no imputation was used. The results based on PP set were used as sensitivity analysis.

All the analyses were performed by SAS version 9.1 (SAS Institute Incorporation, NC, United States).

## Results

### Populations and characteristics

**FIGURE 1:**
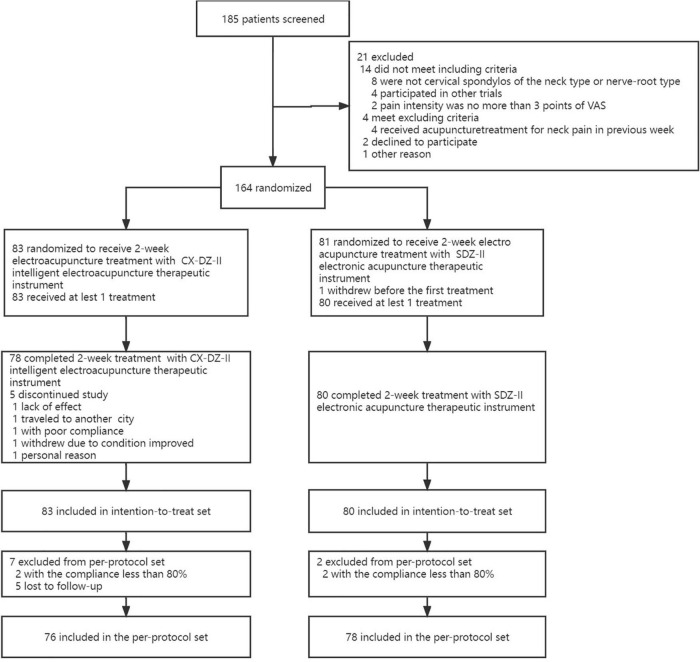
Trial flowchart.

Among 185 participants screened, 164 (83 in the CX-DZ-II group, 81 in the SDZ-II group) participants were randomized (Figure 1). A total of 6 (3.66%) participants dropped out: 1 withdrew before the first treatment and 5 dropped out during the treatment period. For the primary outcome, data were imputed at 4.82 and 0% of participants in the CX-DZ-II and SDZ-II groups. There were no significant differences between groups in terms of gender, age, height, weight, types of CS, coexisting illness, other treatments, participants using other medicine, and the VAS score (Table 1).

**TABLE 1:** Baseline characteristics.

Characteristics	CX-DZ-II (*n* = 83)	SDZ-II (*n* = 81)	*p-*value
**Gender, *n* (%)**			
Male	18 (21.69)	11 (13.75)	0.185
Female	65 (78.31)	69 (86.25)	
Age, mean (SD), yr	47.75 ± 12.19	48.23 ± 13.57	0.813
Height, mean (SD), cm	159.71 ± 6.19	160.00 ± 6.37	0.538
Weight, mean (SD), Kg	58.83 ± 8.75	56.84 ± 8.40	0.141
**Types of CS**			
CS of the neck type	52 (62.65)	59 (73.75)	0.129
CS of the nerve-root type	31 (37.35)	21 (26.25)	
**Coexisting illness, *n* (%)**			
Hypertension	10 (38.46)	1 (5.56)	0.205
Insomnia	4 (15.38)	0 (0.00)	
Others	12 (46.15)	17 (94.44)	
**Other treatments**			
No	83 (100.0)	80 (100.00)	1.0000
Yes	0 (0.00)	0 (0.00)	
**Participants using other medicine, *n* (%)**			
No	63 (75.90)	65 (81.25)	0.406
Yes	20 (24.10)	15 (18.75)	
NSAAs	0 (0.00)	0 (0.00)	
others	20 (100.00)	15 (100.00)	
VAS score, mean (SD)	5.55 (1.47)	5.42 (1.75)	0.613

### Clinical outcomes

The changes from baseline in the VAS throughout 2 weeks were 3.36 in the CX-DZ-II group and 3.23 in the SDZ-II group (difference: 0.17; 95% CI, -0.36 to 0.69, *p* < 0.025 for non-inferiority); similar results were found in the PP set (PPS) (difference: 0.28; 95% CI, -0.38 to 0.93, *p* < 0.025 for non-inferiority). The differences were within the prespecified non-inferiority margin of -0.53, demonstrating that the CX-DZ-II intelligent EA instrument was non-inferiority to the SDZ-II regular electronic stimulator (Table 2).

**TABLE 2:** Primary outcome.

	CX-DZ-II (*n* = 83)	SDZ-II (*n* = 80)	Difference (95% CI)	*P* value
FAS	3.36 (2.92 to 3.79)	3.23 (2.75 to 3.71)	0.17 (-0.36 to 0.69)	0.022
PPS*^a^*	3.53 (3.08 to 3.97)	3.25 (2.76 to 3.74)	0.28 (-0.38 to 0.93)	0.008

FAS, full analysis set; PPS, per-protocol set; CI, confidence intervals.

^a^The number of participants providing data on the VAS score was 76 in the CX-DZ-II group and 78 in the SDZ-II group.

**FIGURE 2:**
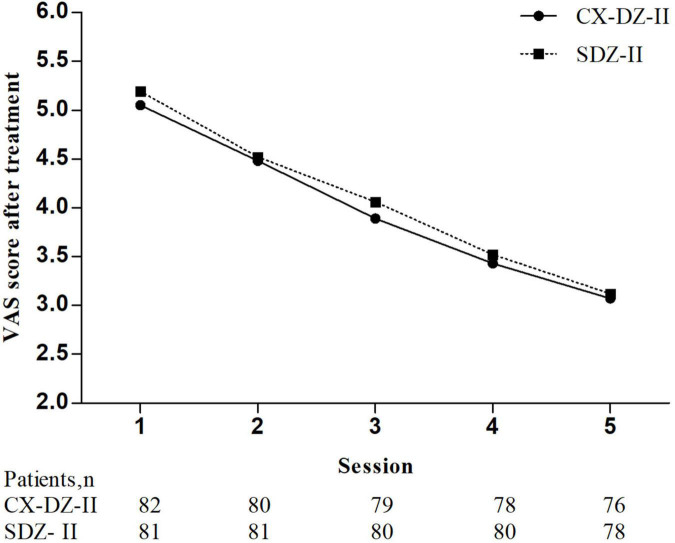
The visual analog scale (VAS) score after treatment at 1 week. *The global test was significant (*p* < 0.0001) and repeated-measures ANOVA with Greenhouse–Geisser correction was used. No significant group differences in 1 week were found (Supplementary Table 3).

No between-group differences were found in the VAS score after treatment at 1 week (all *p* > 0.05, see Supplementary Table 2), overall responders (*p* = 0.869), and drug-usage rate of NSAAs (*p* = 1.000) (Figure 2 and Table 3).

**TABLE 3:** Other secondary outcomes.

Outcome measures*^a^*	CX-DZ-II (*n* = 83)	SDZ-II (*n* = 80)	*p* value
Overall responders, *n* (%)	41 (49.40)	41 (51.25)	0.869
Drug-usage rate of NSAAs, *n* (%)	0 (100)	0 (100)	1.000
Defect rate of the instrument, *n* (%)	6 (7.23)	3 (3.75)	0.496
**Excellent rate of instrument, *n* (%)**			
Score < 16	82 (98.80)	79 (98.75)	1.000
Score > 16	1 (1.20)	1 (1.25)	

*^a^*Statistical analysis set was based on the intention-to-treat population.

In addition, the defect rate of the instrument and the excellent rate of the instrument in the CX-DZ-II intelligent electronic stimulator were similar to the SDZ-II regular electronic stimulator (Table 3).

### Adverse events

No significant difference was observed between the CX-DZ-II group and the SDZ-II group (*p* = 0.169) (Table 4). In terms of non-treatment-related serious AEs, two AEs (got cold) were reported in the CX-DZ-II group, while three AEs (got cold, retinal detachment, and intestinal polypectomy) were reported in the SDZ-II group.

**TABLE 4:** Adverse events related to treatment.*^a^*

AEs	Participant, *n* (%)		*p* value
	CX-DZ-II (*n* = 83)	SDZ-II (*n* = 80)	
**Overall**	9 (10.84)	4 (5.00)	0.169
Fainted during acupuncture	3 (3.61)	1 (1.25)	/
Transient sharp pain	4 (4.82)	3 (3.75)	/
Inserting needles again	2 (2.41)	0 (0.00)	/

AEs, adverse events. *^a^*Adverse events were analyzed based on the full analysis set. AEs with different categories occurring in one participant were defined as different independent AEs.

An AE with multiple occurrences in one participant was defined as a different independent AE.

## Discussion

In this randomized non-inferiority trial of EA for neck pain caused by cervical spondylosis (NPCS), we compared the efficacy and safety of two different EA devices. Findings from the VAS score indicated that the CX-DZ-II intelligent electronic stimulator was non-inferior to the SDZ-II regular electronic stimulator, with a similar safety profile and practicability.

Currently, EA is widely used based on its combined efficacy of manual needles and electric stimulation, especially for pain management. Studies showed that EA could activate sympathetic nerve fibers to enhance the migration of opioid-containing cells to the inflammatory sites or trigger the hypothalamus–pituitary–adrenal axis to decrease cyclooxygenase-2 and then lead to an increase in opioids (Zhang et al., 2014). At the spinal level, EA may also induce several neurotransmitters to inhibit pain, including opioids, 5-hydroxytryptamine, norepinephrine, dopamine, and acetylcholine (Munro, 2007; Yang et al., 2011; Zhang et al., 2012). Of note, functional and structural brain changes due to acupuncture for analgesia were also investigated using neuroimaging techniques (Tu et al., 2021). It has been noticed that acupuncture can reduce brain responses to noxious stimuli in typical regions involved in pain processing, such as the thalamus, insula, and prefrontal cortex (Li et al., 2014; Yan et al., 2020).

Previous studies reported that EA with different frequencies has different analgesic effects (Chen and Han, 1992; Lin et al., 2009). Although EA apparatus can adjust stimulus parameters quantitatively, the accuracy of adjustment is limited. Furthermore, few of them are equipped with networking and visualization systems. The CX-DZ-II intelligent electronic stimulator is designed based on microcomputer net technology and engineering technology. It owns the advantage of accurate parameter adjustment, which is critical to clinical efficacy and safety. More importantly, clinicians can get acupuncture prescriptions from the EBAM and upload the therapeutic information to the cloud database through the terminal unit (Jia et al., 2016; Liang et al., 2017). Thus, we conducted this trial to investigate the efficacy and safety of the CX-DZ-II intelligent electronic stimulator for NP relief in patients with CS.

Neck pain is a common symptom for patients with CS, which has a great impact on people’s quality of life and health. Pain intensity is thought to be one of the primary factors that determine the impact of NP on a person’s overall function and sense of wellbeing. The VAS is a 10-cm scale for pain assessment, which is accessible and easy to administrate. It has been commonly used in pain research and clinical practice, with demonstrated reliability and validity (Li et al., 2007; Moses et al., 2019). Therefore, in this study, a change in the VAS from baseline to the completion of treatment was used as the primary outcome. Moreover, to evaluate the immediate efficacy of the CX-DZ-II intelligent electronic stimulator, the VAS score after each treatment in 1 week has also been reported.

In this study, the changes in the VAS were 3.36 in the CX-DZ-II group vs. 3.23 in the SDZ-II group, within the range of 2.04–4.89 reported in previous trials (Wang, 2012; Wan et al., 2013; Huang, 2015; Garov, 2016). Moreover, findings from the VAS in 1 week indicated that the instant analgesic effect of the CX-DZ-II intelligent electronic stimulator seemed better than the SDZ-II regular electronic stimulator, although no statistical difference was observed. Moreover, similar results of the overall responder and drug-usage rate of NSAAs also verified that the CX-DZ-II intelligent electronic stimulator was non-inferior to the SDZ-II regular electronic stimulator. Although results indicated that the CX-DZ-II intelligent electronic stimulator deserved to be promoted for patients with CS of neck type and nerve root type, the generalizability of the CX-DZ-II regular intelligent electronic stimulator for other CS types still requires further investigation.

The present trial also demonstrated some properties of the CX-DZ-II intelligent electronic stimulator and the SDZ-II regular electronic stimulator. Studies indicate that the sensitivity, manipulation, and therapeutic parameters of devices are critical to the clinical effect and safety of EA. Currently, many EA instruments are equipped with mechanical rotary knobs for parameter adjustment, providing approximate data on electrical frequency and intensity to clinicians (Xu et al., 2016). More importantly, parameter settings of traditional EA instruments need to be reset manually after treatment; otherwise, the excessive current intensity may increase and cause transient discomfort and pain to patients when it is used again. Comparatively, the CX-DZ-II intelligent electronic stimulator is equipped with an ARM-A9 chip, Android 4.0 system, and capacitive touchscreen, which provides conditions for visual and quick parameter adjustment. Moreover, the electrical stimulation parameters can be reset automatically after shutdown, avoiding potential security risks in the next operation. In this study, no between-group differences in the defect rate and the excellent rate were observed, indicating that the CX-DZ-II intelligent electronic stimulator can function, as well as the SDZ-II regular electronic stimulator in terms of performance and operation.

Potential AEs related to treatment should also be noticed. According to previous reports, transient sharp pain is one of the common AEs in trials of EA (Liu et al., 2017, 2021). In this study, similar discomfort appeared in both groups, with no statistical difference. In addition, AEs also involved inserting needles again because of the improper disposal of wire, with no connection with the major structure of the CX-DZ-II intelligent electronic stimulator.

## Conclusion

In conclusion, the effect of the CX-DZ-II intelligent electronic stimulator was non-inferior to the SDZ-II regular electronic stimulator in decreasing the VAS of patients with NPCS. Similarly, the performance of safety and manipulation between groups was consistent. Therefore, we believed that the CX-DZ-II intelligent electronic stimulator, a new device characterized by digitization, networking, and visualization, can provide a promising non-inferior alternative in the treatment of EA for NPCS.

## Limitation

Some limitations of this trial must be acknowledged. First, only the instant effect and safety of the CX-DZ-II intelligent electronic stimulator were verified in this study, and future trials need to assess its persistent effect in the follow-up periods. Second, the degree of participants’ expectations with the results was not evaluated and the pure effect of the CX-DZ-II intelligent electronic stimulator for NPCS needs further assessment. Third, for the CX-DZ-II intelligent electronic stimulator, the specific usage of online functions such as data collection, evidence-based diagnosis, and treatment decision support was not reported because they were not related to the aims of this study.

## Data availability statement

The raw data supporting the conclusions of this article will be made available by the authors, without undue reservation.

## Ethics statement

The studies involving human participants were reviewed and approved by Sichuan Regional Ethics Review Committee, Affiliated to Chengdu University of Traditional Chinese Medicine. The patients/participants provided their written informed consent to participate in this study. Written informed consent was obtained from the individual(s) for the publication of any potentially identifiable images or data included in this article.

## Author contributions

YR and FL: conception and design of the study and critical revision of the manuscript for important intellectual content. HL and YZ: data collection. LC and DL: statistical analysis. LC, DL, and JX: drafting of the manuscript. All authors have contributed to the article and approved the submitted version of the manuscript.

## References

[B1] Bier J. D. Scholten-Peeters W. G. M. Staal J. B. Pool J. Van Tulder M. W. Beekman E. (2018). Clinical practice guideline for physical therapy assessment and treatment in patients with nonspecific neck pain. *Phys. Ther.* 98 162–171. 10.1093/ptj/pzx118 29228289

[B2] Blanpied P. R. Gross A. R. Elliott J. M. Devaney L. L. Clewley D. Walton D. M. (2017). Neck pain: revision 2017. *J. Orthop. Sports Phys. Ther.* 47 A1–A83. 10.2519/jospt.2017.0302 28666405

[B3] Chen X. H. Han J. S. (1992). Analgesia induced by electroacupuncture of different frequencies is mediated by different types of opioid receptors: another cross-tolerance study. *Behav. Brain Res.* 47 143–149. 10.1016/s0166-4328(05)80120-2 1350448

[B4] Chen Z. H. Liang F. R. Yang M. X. Li D. H. Zhang Y. Ren Y. L. (2020). Effect and safety of cx-dz-ii intelligent electroacupuncture therapeutic instrument for neck pain caused by cervical spondylos: study protocol for a randomized controlled trial. *Chin. J. Integr. Med.* 26 375–381. 10.1007/s11655-019-3038-2 31372917

[B5] Chinese Medical Association (2007). *Clinical guidelines for diagnosis and treatment: Pain.* Beijing: People’s Medical Publishing House.

[B6] Comachio J. Oliveira C. C. Silva I. F. R. Magalhães M. O. Marques A. P. (2020). Effectiveness of manual and electrical acupuncture for chronic non-speci?c low back pain: a randomized controlled trial. *J. Acupunct. Meridian Stud.* 13 87–93. 10.1016/j.jams.2020.03.064 32224119

[B7] Corp N. Mansell G. Stynes S. Wynne-Jones G. Morso L. Hill J. C. (2021). Evidence-based treatment recommendations for neck and low back pain across europe: a systematic review of guidelines. *Eur. J. Pain* 25 275–295. 10.1002/ejp.1679 33064878 PMC7839780

[B8] Feng J. Wen C. B. Liang F. R. Luo Y. Chen J. Zhao S. T. (2019). Design of wearable traditional chinese medicine electroacupuncture therapeutic instrument system based on stm32. *J. Tradit. Chin. Med.* 42 64–68. 10.13593/j.cnki.51-1501/r.2019.02.064

[B9] Garov Y. (2016). *The effectiveness and safety of electro-acupuncture and warm acupuncture for the treatment of cervical spondylosis radiculopathy: A randomized clinical trial.* Jiangsu: Nanjing university of Traditional Chinese Medicine.

[B10] Hong W. X. Chen L. Jing J. (2006). *Design of A Novel Intelligent Electroacupuncture with Reinforcement and Reduction Needle Methods in the Traditional Chinese Medicine.* Beijing: Biomedical Engineering.

[B11] Huang X. Z. (2015). *The clinical study on the treatment of cervical spondylotic radiculopathy with Qi ci cervical Jia ji acupoints methods.* Guangdong: Guangzhou university of traditional Chinese medicine.

[B12] Huang Y. Yu M. Kuma A. Klein J. D. Wang Y. Hassounah F. (2021a). Downregulation of let-7 by electrical acupuncture increases protein synthesis in mice. *Front. Physiol*. 12:697139. 10.3389/fphys.2021.697139 34489723 PMC8417904

[B13] Huang C. Z. Li Y. L. Lan X. L. He B. Yang J. Li J. (2021b). Electroacupuncture combined with acupoint catgut embedding for postoperative pain after fistulotomy. *Zhen Ci Yan Jiu* 46 421–425. 10.13702/j.1000-0607.200603 34085467

[B14] Huang J. F. Zheng X. Q. Chen D. Lin J. L. Zhou W. X. Wang H. (2021c). Can acupuncture improve chronic spinal pain? A systematic review and meta-analysis. *Glob. Spine J.* 11 1248–1265. 10.1177/2192568220962440 33034233 PMC8453671

[B15] Jia W. Wen C. Yan X. Chen J. Fu T. (2016). *Intelligence electric acupuncture apparatus system based on wifi.* China: 206133564U.

[B16] Joan Gan C. Y. Chan K. K. Tan J. H. Tan Chor Lip H. Louis Ling L. L. Mohd Azman Z. A. (2021). Smartphone-controlled patch electro-acupuncture versus conventional pain relief during colonoscopy: a randomized controlled trial. *ANZ J. Surg.* 91 E375–E381. 10.1111/ans.16870 33876547

[B17] Kjaer P. Kongsted A. Hartvigsen J. Isenberg-Jørgensen A. Schiøttz-Christensen B. Søborg B. (2017). National clinical guidelines for non-surgical treatment of patients with recent onset neck pain or cervical radiculopathy. *Eur. Spine J.* 26 2242–2257. 10.1007/s00586-017-5121-8 28523381

[B18] Kuo D. T. Tadi P. (2022). *Cervical Spondylosis, Statpearls, StatPearls Publishing Copyright 2022*, Treasure Island: StatPearls Publishing LLC 31855384

[B19] Li J. Zhang J. H. Yi T. Tang W. J. Wang S. W. Dong J. C. (2014). Acupuncture treatment of chronic low back pain reverses an abnormal brain default mode network in correlation with clinical pain relief. *Acupunct. Med.* 32 102–108. 10.1136/acupmed-2013-010423 24280949

[B20] Li L. Liu X. Herr K. (2007). Postoperative pain intensity assessment: a comparison of four scales in chinese adults. *Pain Med.* 8 223–234. 10.1111/j.1526-4637.2007.00296.x 17371409

[B21] Liang F. Shu H. Ren Y. Zeng F. Li J. Guo T. (2017). *Evidence-Based Acupuncture and Moxibustion Meridian-Acupoint Treating and Detecting Instrument Supporting Multiplexed Output and Method of Use Thereof.* Sichuan: Justia

[B22] Lin Y. Peng Y. Yi S. Tang S. Li Z. (2009). Effect of different frequency electroacupuncture on the expression of substance p and β-endorphin in the hypothalamus in rats with gastric distension-induced pain. *Zhen Ci Yan Jiu* 34 252–257. 19916289

[B23] Liu B. Wu J. Yan S. Zhou K. He L. Fang J. (2021). Electroacupuncture vs prucalopride for severe chronic constipation: a multicenter, randomized, controlled, noninferiority trial. *Am. J. Gastroenterol.* 116 1024–1035. 10.14309/ajg.0000000000001050 33273258

[B24] Liu T. Shen H. Yang H. Gao M. (2016). Discussion on the industry standard: electroacupuncture therapy device. *Zhongguo Zhen Jiu* 36 99–101. 26946753

[B25] Liu T. Y. Yang H. Y. Gao M. Xu G. Tang W. C. (2010). Single-acupoint electroacupuncture based on traditional acupuncture becomes true. *Zhen Ci Yan Jiu* 35 384–387. 21235069

[B26] Liu Z. Liu Y. Xu H. He L. Chen Y. Fu L. (2017). Effect of electroacupuncture on urinary leakage among women with stress urinary incontinence: a randomized clinical trial. *JAMA* 317 2493–2501. 10.1001/jama.2017.7220 28655016 PMC5815072

[B27] Luo M. (2014). *Clinical Observation on Lumbar Intervertebral Disc Herniation Treated by Electro-Acupunture of Jiaji Points Combined with Mckenzie Therapy.* Guangzhou: University Traditional Chinese Medicine.

[B28] Lv Y. Tian W. Chen D. Liu Y. Wang L. Duan F. (2018). The prevalence and associated factors of symptomatic cervical spondylosis in chinese adults: a community-based cross-sectional study. *BMC Musculoskelet. Disord.* 19:325. 10.1186/s12891-018-2234-0 30205836 PMC6134586

[B29] Mao J. J. Liou K. T. Baser R. E. Bao T. Panageas K. S. Romero S. A. D. (2021). Effectiveness of electroacupuncture or auricular acupuncture vs usual care for chronic musculoskeletal pain among cancer survivors: the peace randomized clinical trial. *JAMA Oncol.* 7 720–727. 10.1001/jamaoncol.2021.0310 33734288 PMC7974834

[B30] Moses M. J. Tishelman J. C. Stekas N. Jevotovsky D. S. Vasquez-Montes D. Karia R. (2019). Comparison of patient reported outcome measurement information system with neck disability index and visual analog scale in patients with neck pain. *Spine* 44 E162–E167. 10.1097/BRS.0000000000002796 30015716

[B31] Munro G. (2007). Dopamine d(1) and d(2) receptor agonism enhances antinociception mediated by the serotonin and noradrenaline reuptake inhibitor duloxetine in the rat formalin test. *Eur. J. Pharmacol.* 575 66–74. 10.1016/j.ejphar.2007.07.062 17725928

[B32] Safiri S. Kolahi A. A. Hoy D. Buchbinder R. Mansournia M. A. Bettampadi D. (2020). Global, regional, and national burden of neck pain in the general population, 1990-2017: systematic analysis of the global burden of disease study 2017. *BMJ* 368:m791. 10.1136/bmj.m791 32217608 PMC7249252

[B33] Seo S. Y. Lee K. B. Shin J. S. Lee J. Kim M. R. Ha I. H. (2017). Effectiveness of acupuncture and electroacupuncture for chronic neck pain: a systematic review and meta-analysis. *Am. J. Chin. Med.* 45 1573–1595. 10.1142/S0192415X17500859 29121797

[B34] Shen H. Liu T. Y. Yang H. Y. (2016). Review of application technology of electroacupuncture instrument and new idea of its development. *Shanghai J. Acupunct. Moxibustion* 35 1016–1020. 10.13460/j.issn.1005-0957.2016.08.1016

[B35] Su Z. Hu L. Cheng J. Klein J. D. Hassounah F. Cai H. (2016). Acupuncture plus low-frequency electrical stimulation (acu-lfes) attenuates denervation-induced muscle atrophy. *J. Appl. Physiol.* 120 426–436. 10.1152/japplphysiol.00175.2015 26679610 PMC4754622

[B36] Tang S. Luo X. Y. Shi Q. D. Lin Y. P. Lai L. He L. (2014). Observations on the efficacy of electroacupuncture at huatuo jiaji points in treating cervical spondylosis *Shanghai J. Acu-Mox.* 33 840–842. 10.13460/j.issn.1005-0957.2014.09.0840

[B37] Tu Y. Cao J. Bi Y. Hu L. (2021). Magnetic resonance imaging for chronic pain: diagnosis, manipulation, and biomarkers. *Sci. Chin. Life Sci.* 64 879–896. 10.1007/s11427-020-1822-4 33247802

[B38] Vogt M. T. Cawthon P. M. Kang J. D. Donaldson W. F. Cauley J. A. Nevitt M. C. (2006). Prevalence of symptoms of cervical and lumbar stenosis among participants in the osteoporotic fractures in men study. *Spine* 31 1445–1451. 10.1097/01.brs.0000219875.19688.a6 16741453

[B39] Wan B. J. Huang W. Zhang Y. X. Zhang H. S. (2013). Influence of electroacupuncture with penetration needling method on comprehensive pain score in patients with cervical spondylotic radiculopathy. *Zhongguo Zhen Jiu* 33 407–410. 23885612

[B40] Wang W. (2012). *The clinical study by randomized controlled trials on Qi ci cervical Jia ji acupoints in treatment of cervical spondylosis radiculopathy.* Sichuan: Chengdu university of Traditional Chinese Medicine.

[B41] Wang Z. J. (2019). *Research on Traceability Method and Development of the Calibration Device for Electro-Acupuncture Treatment Instrument.* Zhejiang: Association of Pacific Rim Universities.

[B42] Xu F. P. Chen Z. L. Guo Y. (2016). Reports on electroacupuncture device parameters and analysis of its measured output frequencies. *Shanghai J. Acupunct. Moxibustion* 35 1139–1142.

[B43] Yan C. Q. Huo J. W. Wang X. Zhou P. Zhang Y. N. Li J. L. (2020). Different degree centrality changes in the brain after acupuncture on contralateral or ipsilateral acupoint in patients with chronic shoulder pain: a resting-state fmri study. *Neural Plast.* 2020:5701042. 10.1155/2020/5701042 32377180 PMC7197008

[B44] Yang E. J. Koo S. T. Kim Y. S. Lee J. E. Hwang H. S. Lee M. S. (2011). Contralateral electroacupuncture pretreatment suppresses carrageenan-induced inflammatory pain via the opioid-mu receptor. *Rheumatol. Int.* 31 725–730. 10.1007/s00296-010-1364-y 20130880

[B45] Yang Y. X. Yong M. C. (2009). Safety problems and countermeasures of electroacupuncture instruments. *Zhongguo Zhen Jiu* 29 339–341. 19565747

[B46] Zhang R. Lao L. Ren K. Berman B. M. (2014). Mechanisms of acupuncture-electroacupuncture on persistent pain. *Anesthesiology* 120 482–503. 10.1097/ALN.0000000000000101 24322588 PMC3947586

[B47] Zhang Y. Zhang R. X. Zhang M. Shen X. Y. Li A. Xin J. (2012). Electroacupuncture inhibition of hyperalgesia in an inflammatory pain rat model: involvement of distinct spinal serotonin and norepinephrine receptor subtypes. *Br. J. Anaesth.* 109 245–252. 10.1093/bja/aes136 22628394 PMC3393079

