# Supplementary material for: The CX-DZ-II intelligent electronic stimulator for neck pain caused by cervical spondylosis: A two-center, randomized, controlled, and non-inferiority trial
Source: Front Neurosci. 2022 Jul 28;16:910574. doi: 10.3389/fnins.2022.910574 (PMC9366011; doi:10.3389/fnins.2022.910574)
Supplement: Supplementary file 1 [file Table_1.DOCX]

| ***Supplementary Table1*. The operating performance scale of the instrument** | | |
| --- | --- | --- |
| **Category** | **Grade** | **Explanation** |
| Operability of instrument | 1 | It’s very easy to operate |
|  | 2 | It's easy to operate |
|  | 3 | It's a little bit to operate |
|  | 4 | It's difficult to operate |
|  | 5 | It's very difficult to operate |
| Sensitivity of parameter adjustment | 1 | It’s very quick to respond |
|  | 2 | It’s quick to respond without use disorders |
|  | 3 | It’s slow to respond with little use disorders |
|  | 4 | It’s slow to respond with severe use disorders, but can be used for treatment |
|  | 5 | It’s slow to respond and can not be used for treatment |
| Stability of voltage output | 1 | It’s stable |
|  | 2 | It’s a little bit unstable without use disorders |
|  | 3 | It’s unstable with little use disorders |
|  | 4 | It’s unstable with severe use disorders, but can be used for treatment |
|  | 5 | It’s unstable and can not be used for treatment |
| Instrument fault | 1 | None |
|  | 2 | It has a little instrument fault without use disorders |
|  | 3 | It has some instrument fault with little use disorders |
|  | 4 | It has some instrument faults with severe use disorders but can be used for treatment |
|  | 5 | It has severe instrument fault and can not be used for treatment |

| ***Supplementary Table2*. VAS score after treatment at week1, mean (SD)^a^** | | | | |
| --- | --- | --- | --- | --- |
|  | **CX-DZ-II (n=83)** | **SDZ-II (n=80)** | **Difference (95% CI)** | *p* ***value*** |
| VAS score after 1^st^ treatment^b^ | 5.05(1.26) | 5.19(1.26) | -0.15(-0.54,0.24) | 0.462 |
| VAS score after 2^nd^ treatment^c^ | 4.48 (1.31) | 4.52(1.43) | -0.04(-0.46,0.39) | 0.855 |
| VAS score after 3^rd^ treatment^d^ | 3.89(1.33) | 4.06(1.45) | -0.17(-0.60,0.26) | 0.434 |
| VAS score after 4^th^ treatment^e^ | 3.43(1.27) | 3.52(1.47) | -0.09(-0.51,0.34) | 0.684 |
| VAS score after 5^th^ treatment^f^ | 3.07(1.17) | 3.12(1.44) | -0.05(-0.46,0.35) | 0.794 |

^a^Statistical analyses set were based on intention-to-treat population.

^b^The number of participants providing data of VAS was 82 in CX-DZ-II group and 81 in SDZ-II group.

^c^The number of participants providing data of VAS was 80 in CX-DZ-II group and 81 in SDZ-II group.

^d^The number of participants providing data of VAS was 79 in CX-DZ-II group and 80 in SDZ-II group.

^e^The number of participants providing data of VAS was 78 in CX-DZ-II group and 81 in SDZ-II group.

^f^The number of participants providing data of VAS was 76 in CX-DZ-II group and 78 in SDZ-II group.

| ***Supplementary Table3*. Instrument fault list of two groups during the whole study** | | |
| --- | --- | --- |
| **Instrument fault, n(%)** | **CX-DZ-II group (n=83)** | **SDZ-II group (n=80)** |
| Screen flicker | 1(1.20%) | 0 |
| Damage of chip | 1(1.20%) | 0 |
| Inserted needles were pulled out because of the improper disposal of wire | 2(2.41%） | 0 |
| Transient increase of electricity without expectation | 2(2.41%） | 2(2.50%) |
| The intensity of electricity could not be adjusted | 0 | 1(1.25%) |
